# Unveiling genetic links between gut microbiota and asthma: a Mendelian randomization

**DOI:** 10.3389/fmicb.2024.1448629

**Published:** 2024-09-20

**Authors:** XuWen Zheng, MaoBing Chen, Yi Zhuang, Liang Zhao, YongJun Qian, ChengCheng Shi

**Affiliations:** Emergency Department, Wujin People’s Hospital Affiliated with Jiangsu University, Wujin Clinical College of Xuzhou Medical University, Changzhou, Jiangsu, China

**Keywords:** asthma, gut microbiota, Linkage Disequilibrium Score Regression, Mendelian randomization, meta-analysis

## Abstract

**Background:**

Multiple studies suggest a potential connection between the gut microbiome and asthma. Our objective is to use advanced genetic and metagenomic techniques to elucidate the causal relationships and underlying mechanisms between gut microbiota and asthma.

**Methods:**

The study utilized comprehensive Linkage Disequilibrium Score Regression (LDSC) and Mendelian randomization (MR) analyses to examine the relationship between 119 gut microbiota genera and asthma, using publicly accessible genome-wide association studies (GWAS). The meta-analysis synthesized summary effect estimates obtained from LDSC, forward MR, and reverse MR. The MiBioGen collaboration, involving 18,340 individuals, identified genetic variations associated with gut bacteria. Asthma data were collected from the UK Biobank, FinnGen, and GERA, encompassing a total of 82,060 cases and 641,049 controls.

**Results:**

LDSC analysis revealed significant negative genetic correlations between asthma and *RuminococcaceaeUCG004* (Rg = −0.55, *p* = 7.66 × 10^−5^) and *Subdoligranulum* (Rg = −0.35, *p* = 3.61 × 10^−4^). Forward MR analysis suggested associations between *Butyricicoccus* (OR = 0.92, *p* = 0.01), *Turicibacter* (OR = 0.95, *p* = 0.025), *Butyrivibrio* (OR = 0.98, *p* = 0.047), and reduced asthma risk. Conversely, *Coprococcus2* (OR = 1.10, *p* = 0.035) and *Roseburia* (OR = 1.07, *p* = 0.039) were associated with increased risk. Reverse MR analysis indicated significant associations between genetically predicted asthma and *Eubacteriumxylanophilumgroup* (Beta = −0.08, *p* = 9.25 × 10^−7^), *LachnospiraceaeNK4A136group* (Beta = −0.05, *p* = 1.26 × 10^−4^), and *Eisenbergiella* (Beta = 0.06, *p* = 0.015, Rg_*P* = 0.043).

**Conclusion:**

The findings underscore significant genetic correlations and causal relationships between specific gut microbiota and asthma. These insights highlight the potential of gut microbiota as both markers and modulators of asthma risk, offering new avenues for targeted therapeutic strategies.

## 1 Introduction

Asthma affects approximately 300 million people worldwide and its prevalence has been increasing over the past few decades, especially in industrialized countries (Boulet et al., 2019). The condition is more common in children, with symptoms often beginning in early childhood, but it can develop at any age (Martinez and Vercelli, 2013). Clinically, asthma is characterized by recurring bouts of coughing, chest tightness, shortness of breath, and wheezing, often occurring at night or in the early morning (Balmes, 1993). Asthma also poses significant health burdens, including frequent hospital visits, decreased quality of life, and even death in severe cases (Fanta, 2002). Environmental factors like air pollution and tobacco smoke exacerbate asthma symptoms, such as more frequent hospital visits and worsening of symptoms like coughing, chest tightness, and shortness of breath. Additionally, the study found a correlation between increased exposure to environmental pollutants and elevated levels of inflammatory biomarkers in the lungs of asthmatic patients, suggesting a direct link between environmental triggers and asthma exacerbation (Comhair et al., 2011). The complex disease is influenced by genetic, environmental, and immunological factors. Traditionally considered a Th2-mediated allergic disorder, recent research indicates that asthma also involves Th17 responses and non-allergic pathways (Holgate, 2008). Environmental triggers such as allergens, viruses, and pollutants interact with genetic susceptibilities to cause airway inflammation and remodeling (Holtzman, 2012). This multifaceted pathogenesis underscores the importance of tailored therapeutic approaches targeting specific mechanisms underlying asthma.

Among the most promising domains in asthma research is the involvement of the microbiome as a potential environmental contributor. Viruses and fungi have been reported to be correlated with various allergic conditions. For example, temperate gut phage taxa, particularly the joint abundances of 19 caudoviral families, were associated with later development of asthma (Leal Rodríguez et al., 2024); Proteases and chitin, produced by fungi such as *Alternaria*, *Aspergillus*, and *Cladosporium* were capable of inducing type 2 immune responses via toll-like receptor 4 (Zheng and Dang, 2023). Insights into the gut microbiota’s role in modulating immune responses have opened another avenue for understanding asthma’s etiology. Studies suggest that the gut microbiota can influence immune responses and inflammation, which are critical in asthma pathogenesis (Frati et al., 2018; Barcik et al., 2020). The pathogenesis of asthma in relation to gut microbiota involves several mechanisms. For example, gut microbiota produce metabolites such as short-chain fatty acids (SCFAs), which can modulate immune responses and inflammation. Dysbiosis, or an imbalance in gut microbiota, may lead to altered production of these metabolites, contributing to asthma (Wang et al., 2018). Despite the growing body of evidence linking gut microbiota to asthma, several gaps remain. Most studies have focused on associations rather than causal mechanisms, and the specific pathways through which gut microbiota influence asthma are not fully understood. Our study aims to fill these gaps by employing Mendelian randomization (MR) to elucidate the causal relationships and underlying mechanisms between gut microbiota and asthma.

Understanding the genetic associations between gut microbiota and asthma is crucial as it can provide novel insights into the pathophysiology and potential treatment strategies for asthma. Asthma remains a significant public health concern, and traditional treatments often do not address the underlying causes of the disease. By focusing on the gut-lung axis (GLA), our study aims to uncover how gut microbiota composition and diversity influence asthma development and severity.

## 2 Materials and methods

### 2.1 Study design

**Figure 1:**
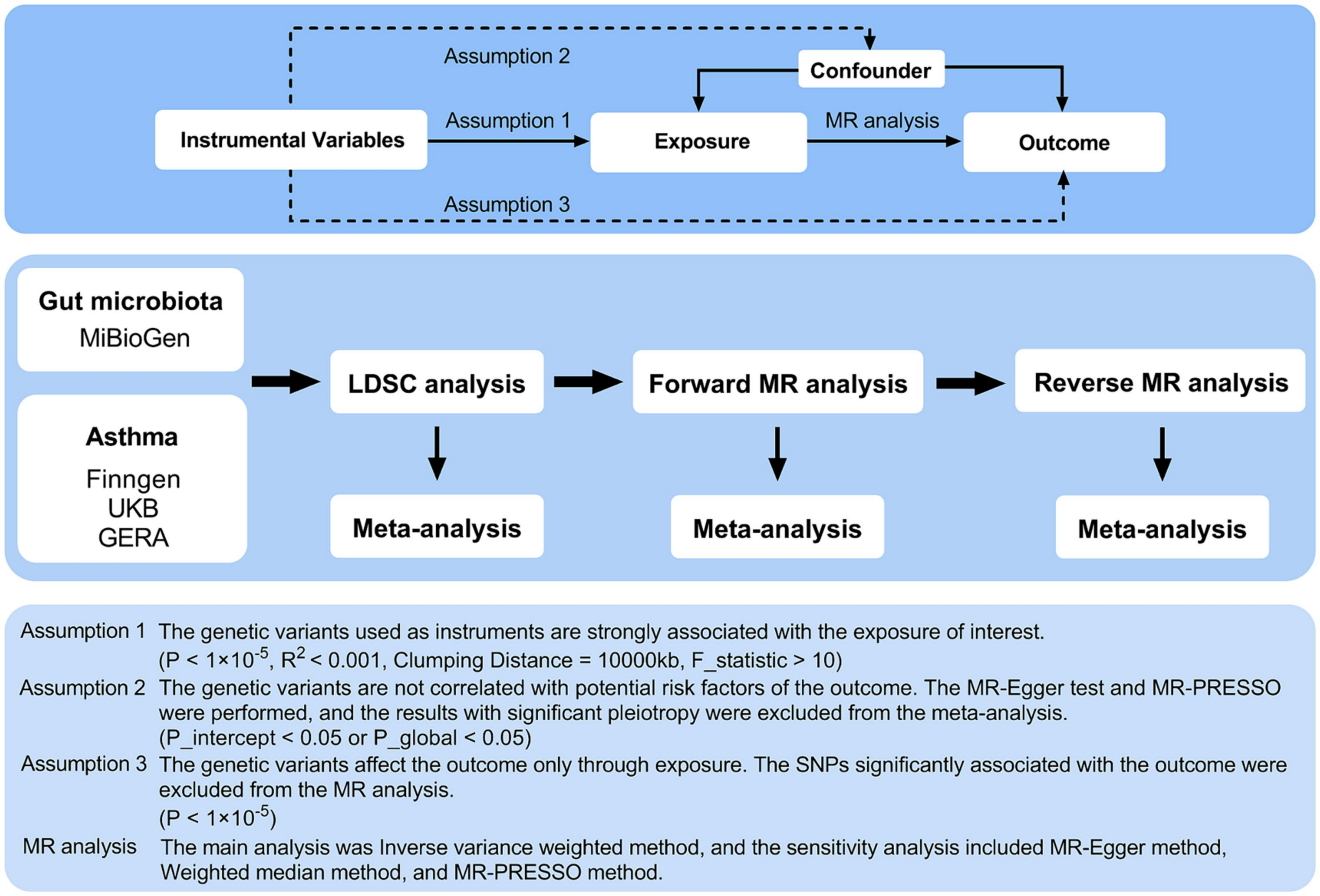
Three assumptions of MR analysis and overview of the study design. MR, Mendelian randomization; GERA, Genetic Epidemiology Research on Aging; LDSC, Linkage Disequilibrium Score Regression; MR-PRESSO, MR pleiotropy residual sum and outlier; SNPs, single nucleotide polymorphisms.

The detailed structure of this investigation is shown in Figure 1. The study examined the relationship between 119 genera of gut microbiota and asthma through comprehensive analyses using Linkage Disequilibrium Score Regression (LDSC) and MR. The application of instrumental variables (IVs) in multiple regression analysis relies on three key assumptions: (1) the genetic variants used as instruments must have a significant association with the exposure being studied; (2) these variants must be unrelated to any potential confounding factors that could impact the outcome; and (3) the influence of the genetic variants on the outcome must be solely through the exposure variable (Burgess and Thompson, 2015). A meta-analysis was conducted to evaluate asthma using multiple data sources, incorporating the overall impact estimates from LDSC, forward MR, and reverse MR. The analysis was conducted using summary-level data from published genome-wide association studies (GWASs) and the analytic process was in accordance with the STROBE-MR guidelines (Skrivankova et al., 2021). Consent was obtained from all participants in all studies included in this research, as approved by the appropriate institutional review boards and ethics committees.

### 2.2 Instrumental variable selection

The MiBioGen team conducted the most extensive genome-wide meta-analysis to date to identify genetic variants associated with the composition of the intestinal microbiota (Kurilshikov et al., 2021). This investigation included 18,340 participants, the majority of whom were of European descent, from 24 cohorts (*n* = 13,266). The lowest taxonomic level examined was genus, revealing 131 genera with a mean abundance of more than 1%, including 12 unidentified genera. Consequently, the analysis encompassed 119 taxa at the genus level. When using a threshold of *p* < 5 × 10^−8^ and applying a stringent linkage disequilibrium (LD) clumping setting with a 10,000 kb distance and *r*^2^ < 0.001 between instrumental variables (IVs), we found that only 16 genera had total of 17 SNPs meeting these criteria. To satisfy the first MR analysis assumption, the gut microbiota identified by GWAS was subjected to a significance threshold of *p* < 1 × 10^−5^ (Sanna et al., 2019). However, employing a threshold of *p* < 5 × 10^−8^ is still a significant association between the gut microbiome and asthma. To prevent weak IVs, the *F*-statistic of each intestinal microbiota (*F* = beta^2^/se^2^) was calculated; those with an *F*-statistic of less than 10 were deemed weak and excluded (Bowden et al., 2019; Xie et al., 2023). SNPs in the exposure and outcome datasets were harmonized to match reference and alternative alleles, thereby eliminating mismatched SNPs to minimize discrepancies. Additionally, the MR analysis excluded ambiguous palindromic SNPs with minor allele frequencies of approximately 0.5. To identify significant pleiotropy, MR estimates with pleiotropy were excluded from the meta-analysis under the second assumption using the MR-Egger intercept test and the MR pleiotropy residual sum and outlier (MR-PRESSO) test (*p* for intercept <0.05 or *p* for global test <0.05). To ensure precise causal conclusions, SNPs substantially associated with the outcome (*p* < 1 × 10^−5^) were excluded from the MR analysis for the third assumption. A comprehensive list of the IVs associated with each taxon in the gut microbiota is provided in Supplementary Table 1.

### 2.3 Asthma data sources

Three primary sources provided summary-level data on asthma: the UKB GWAS (Sudlow et al., 2015); the FinnGen GWAS Release 10 (Kurki et al., 2023); and the Genetic Epidemiology Research on Aging (GERA) (Guindo-Martínez et al., 2021). The combined sample comprised 641,049 European-ancestry controls and 82,060 cases. The UKB GWAS included 500,000 participants, collected between 2006 and 2010, in a major multicenter cohort study (Sudlow et al., 2015). We used the European ancestry summary data from the Lee lab’s GWAS, where phecode 495 was used to define asthma outcomes. Kurki et al. described the FinnGen GWAS as a comprehensive national genetic research project that integrates genomic data with electronic health information (Kurki et al., 2023). The International Classification of Diseases, Ninth Revision (ICD-9: 493), and Tenth Revision (ICD-10: J45, J46) codes were used to identify clinical endpoints such as asthma-related hospital admissions, emergency room visits, and prescribed medications for asthma management. The study highlighted that individuals with certain genetic markers were more likely to experience severe asthma symptoms, frequent exacerbations, and a higher need for corticosteroids or beta-agonists, indicating the genetic predisposition’s role in asthma’s clinical course. In the GERA data, asthma diagnoses also followed ICD-9 (493) (Guindo-Martínez et al., 2021). All the sources of outcome were applied exclusion criteria to rule out participants with known comorbid infections, malignant diseases, or other miscellaneous conditions that could potentially confound the associations being studied. Table 1 provides detailed summaries of the studies utilized.

**Table 1:** Detailed information on used summary-level data.

Exposure or outcome	Consortium	Participants included in analysis	Males (%)	Average age (years)	Adjustments	Eligibility criteria	Web source
Gut microbiota	MiBioGen	18,340 multiple-descent individuals	44	55 (32–89)	Age, sex, technical covariates and genetic principal components		https://mibiogen.gcc.rug.nl/
Asthma	Finngen	46,684 cases and 219,734 controls of European ancestry	44	53 ± 18	Sex, age, genotyping batch and 10 principal components	ICD-10: J45, J46; ICD-9: 493	https://r10.finngen.fi/
	UKB	26,167 cases and 373,887 controls of European ancestry	48	55.1 ± 7.6	Age, sex, age*sex, age^2^, age^2^*sex, first 10 genetic principal components	Phecode: 495	https://www.leelabsg.org/resources
	GERA	9,209 cases and 47,428 controls of European ancestry	42	63 (19 to over 100)	Seven derived principal components, sex, and age	ICD-9: 493	http://cg.bsc.es/gera_summary_stats/

### 2.4 Statistical analysis

Using LDSC, we examined the genetic correlation between the gut microbiota and asthma. The GWAS summary data were refined using HapMap3 references, which involved eliminating non-SNP variants such as insertions/deletions (indels), ambiguous SNPs, duplicated SNPs, and those with a minor allele frequency below 0.01. LDSC can quantify genetic connections using GWAS summary data by assessing the relationship between LD and test statistics, determining if observed inflation is caused by genuine polygenic signals or other biases (Bulik-Sullivan B. et al., 2015). This approach is unaffected by sample overlap (Bulik-Sullivan B. K. et al., 2015). Genetic covariance is calculated by performing a regression analysis on the products obtained by multiplying the *z*-scores of variants associated with Trait 1 by those associated with Trait 2, after multiplying the *z*-scores by the LD score (Wielscher et al., 2021). The genetic relationship becomes evident after applying SNP-heritability to this covariance. The genetic relationship between gut microbiota and asthma was estimated by integrating data from three different datasets using fixed-effects meta-analysis.

For causal analysis, the primary MR estimate was calculated using the inverse-variance weighted (IVW) method within a random-effects model. IVW method is best used when the MR assumptions are believed to hold true across all genetic variants. It provides the most precise estimate when there is no horizontal pleiotropy (Burgess and Thompson, 2015). To evaluate the presence of horizontal pleiotropy and confirm the reliability of the data, we performed three sensitivity analyses: weighted median, MR-Egger, and MR-PRESSO. The weighted median method is particularly useful when there is concern that some genetic variants may be invalid instruments due to pleiotropy. It provides a robust estimate that is less sensitive to invalid instruments compared to the IVW method (Bowden et al., 2016). MR-Egger is particularly useful when there is concern about directional pleiotropy. It provides a more conservative estimate and tests for the presence of pleiotropy through the intercept term. If the intercept is significantly different from zero, this indicates the presence of directional pleiotropy (Burgess and Thompson, 2017). MR-PRESSO is best used when there is evidence or suspicion of pleiotropy. It improves the reliability of causal estimates by removing the influence of outlier variants that violate the exclusion restriction assumption (Verbanck et al., 2018). To assess SNP heterogeneity, we used the Cochran *Q* value. Horizontal pleiotropic effects were identified using the MR-Egger intercept test. The estimates derived from the IVW and sensitivity analyses were combined using fixed-effects meta-analysis. Exposures represented by fewer than four SNPs were excluded, as MR-PRESSO requires a minimum of four instrumental SNPs. Additionally, the meta-analysis excluded estimates indicating significant pleiotropy, defined by a *p*-value less than 0.05 for either the intercept test or the global test. The power of MR analysis was estimated using an online tool (Brion et al., 2013).

Bonferroni’s correction was applied separately to both LDSC and MR analyses in the meta-analyses to minimize the false discovery rate (Curtin and Schulz, 1998). LDSC correlations with *p*-values between 6.41 × 10^−4^ (0.05/78) and 0.05 were suggestive, while those with *p*-values less than 6.41 × 10^−4^ were significant. MR associations were suggestive if IVW *p*-values were between 4.20 × 10^−4^ (0.05/119) and 0.05, and significant if *p*-values were less than 4.20 × 10^−4^ or if both IVW and LDSC *p*-values were less than 0.05. The statistical analyses were performed using R software (version 4.3.1) and included the use of the GenomicSEM, meta, and TwoSampleMR packages.

## 3 Results

### 3.1 LDSC analysis

**Figure 2:**
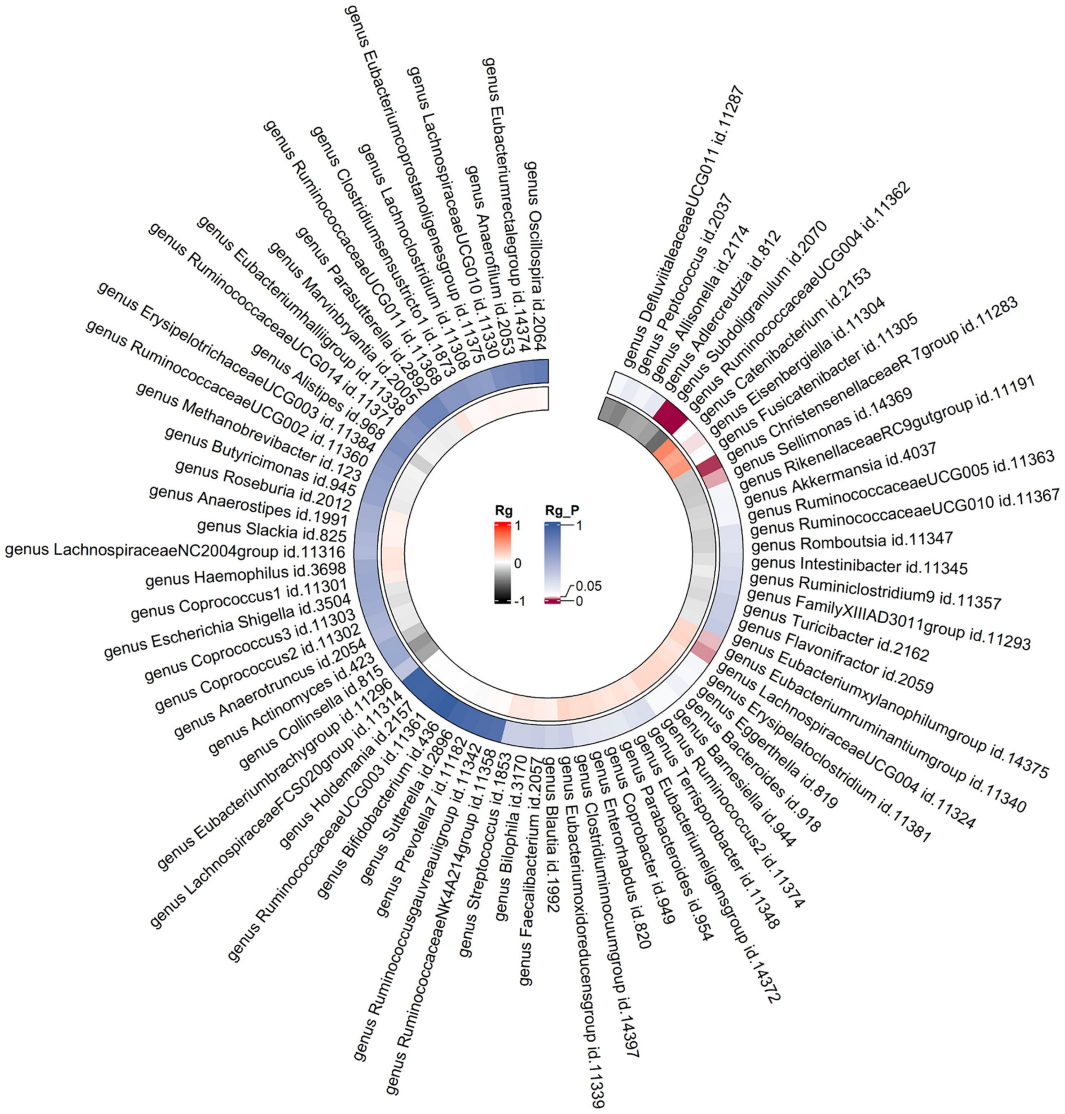
Circular heat map of meta-analysis of genetic correlation between gut microbiota and asthma. Rg, estimate of genetic correlation; Rg_*P*, *p*-value for Rg.

Constraints such as low heritability and limited sample sizes restrict the suitability of certain bacterial taxa for LDSC analysis. Through a meta-analysis of LDSC, we assessed the genetic correlation between 78 gut microbes and asthma (Figure 2). As shown in Table 2, significant negative genetic correlations were identified for *RuminococcaceaeUCG004* (Rg = −0.55, *p* = 7.66 × 10^−5^) and *Subdoligranulum* (Rg = −0.35, *p* = 3.61 × 10^−4^) with asthma. Additionally, *ChristensenellaceaeR7group* and *Sellimonas* showed a suggestive negative correlation, whereas *LachnospiraceaeUCG004*, *Eubacteriumruminantiumgroup*, and *Eisenbergiella* displayed a suggestive positive correlation with asthma. No heterogeneity or mild heterogeneity was observed across most of the results. Supplementary Table 2 contains comprehensive details of all genetic correlation findings.

**Table 2:** Meta-analysis of genetic correlation between gut microbiota and asthma from three large databases.

Exposure	Rg	Rg_Se	Rg_*P*	*P*_heterogeneity
Genus *RuminococcaceaeUCG004*	−0.549	0.139	7.66E−05	0.692
Genus *Subdoligranulum*	−0.346	0.097	3.61E−04	0.746
Genus *ChristensenellaceaeR 7group*	−0.186	0.068	0.006	0.547
Genus *LachnospiraceaeUCG004*	0.217	0.097	0.025	0.514
Genus *Sellimonas*	−0.194	0.090	0.030	0.922
Genus *Eubacteriumruminantiumgroup*	0.202	0.096	0.035	0.561
Genus *Eisenbergiella*	0.459	0.227	0.043	0.686

### 3.2 Forward MR analysis

**Figure 3:**
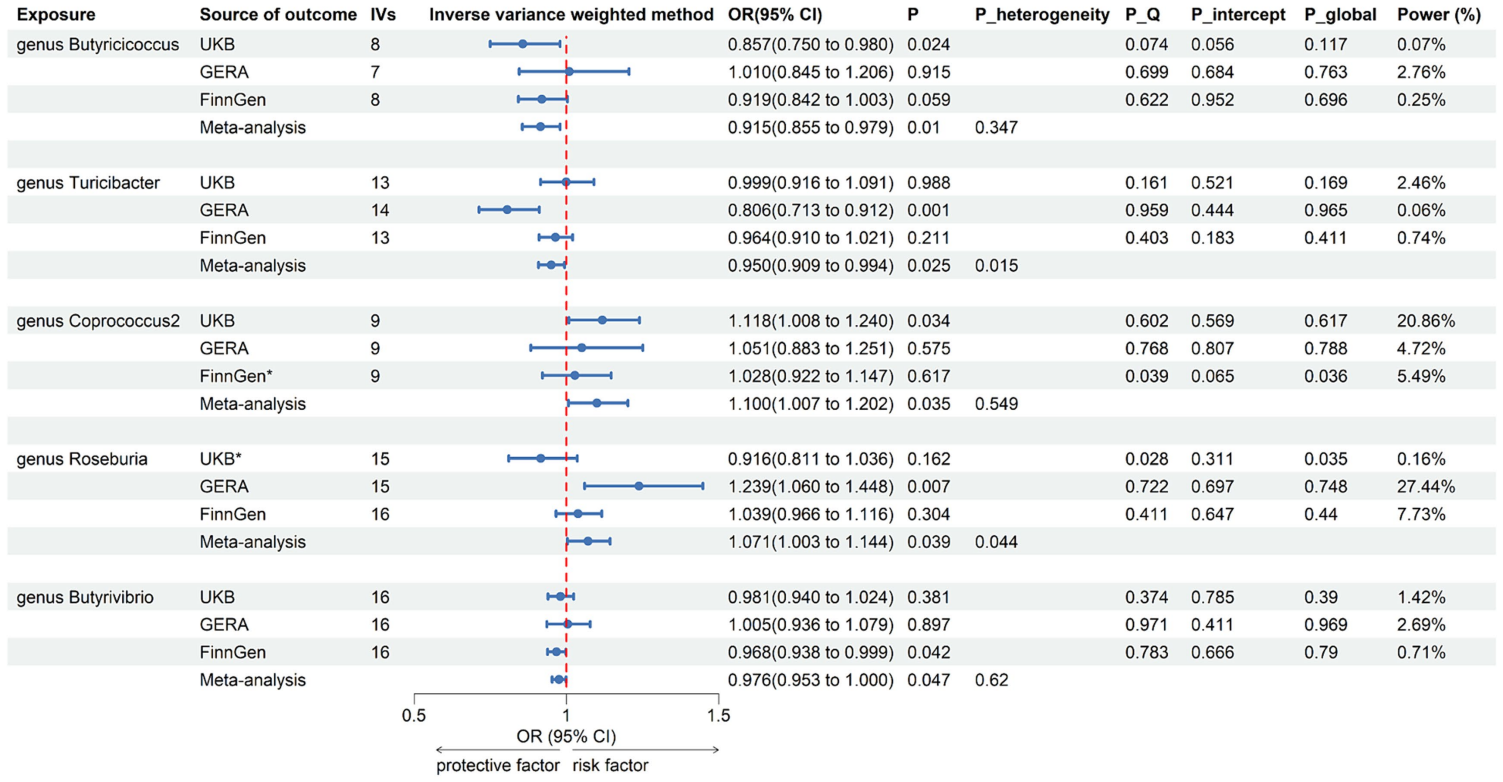
Forest plot of associations in forward MR analysis. IVs, instrumental variables; CI, confident interval; *P*_heterogeneity, *p*-value of heterogeneity for meta-analysis; *P*_Q, *p*-value for Cochran *Q* test; *P*_intercept, *p*-value for MR-Egger intercept test; *P*_global, *p*-value for Global test; *, excluded from the meta-analysis due to SNPs less than 4 or significant pleiotropy.

**Figure 4:**
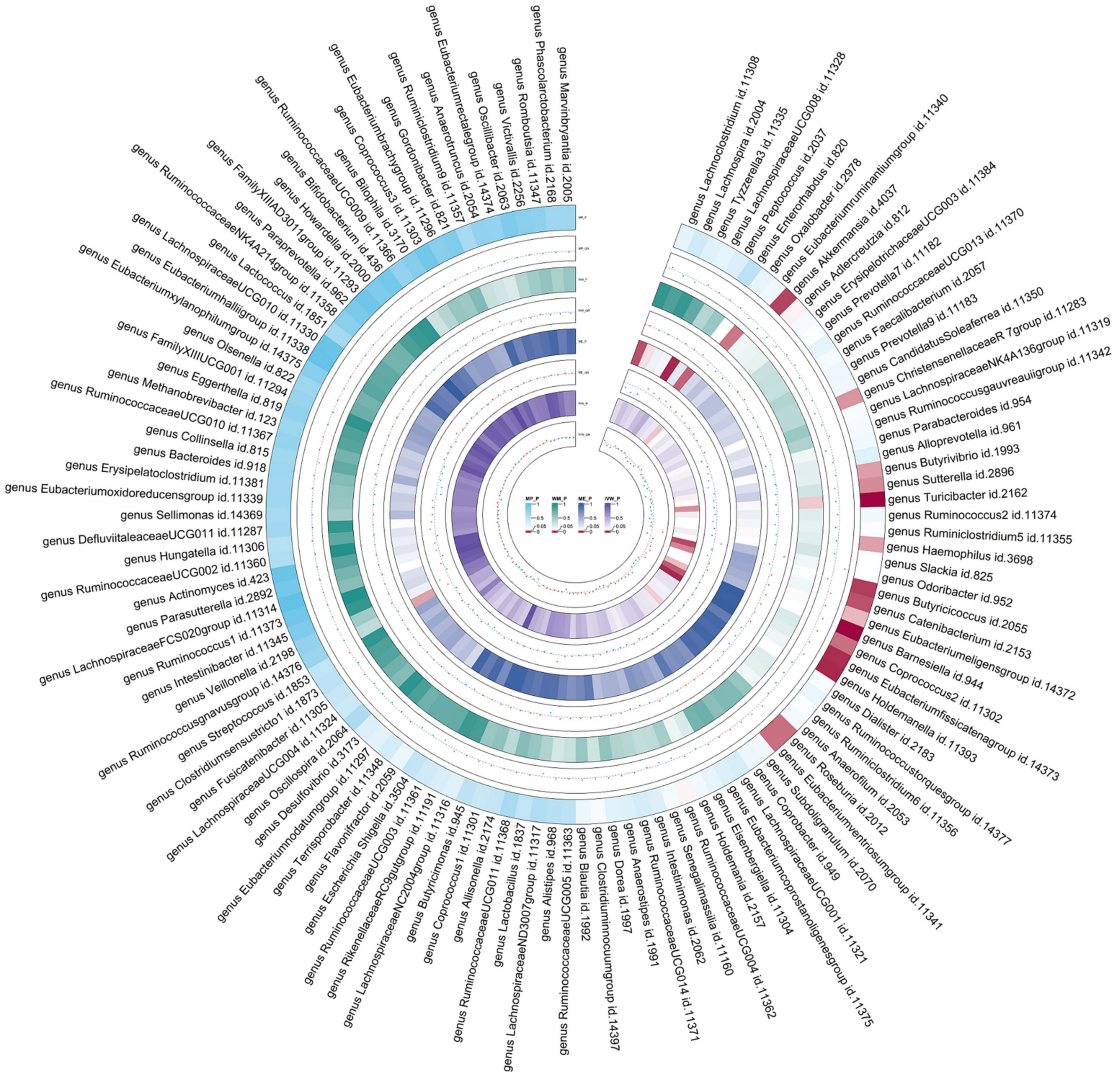
Circular heat map of meta-analysis of forward MR analysis between gut microbiota and asthma. IVW, Inverse-Variance Weighted; ME, MR-Egger; WM, Weighted median; MP, MR-PRESSO. The color variations represented the size of the *p*-value. The scatter plots reflect OR, with OR > 1 labeled red and OR < 1 labeled green.

Following the selection of instrumental variables, 119 meta-analyses were performed, revealing that five bacterial genera had suggestive associations with asthma (Supplementary Table 3). According to the IVW method, genetically predicted *Butyricicoccus* (OR = 0.92, 95% CI 0.86–0.98; *p* = 0.01), *Turicibacter* (OR = 0.95, 95% CI 0.91–0.99; *p* = 0.025), and *Butyrivibrio* (OR = 0.98, 95% CI 0.95–0.99; *p* = 0.047) were associated with a reduced risk of asthma. Conversely, *Coprococcus2* (OR = 1.10, 95% CI 1.01–1.20; *p* = 0.035) and *Roseburia* (OR = 1.071, 95% CI 1.003–1.144; *p* = 0.039) were associated with an increased risk (Figure 3). All sensitivity analyses supported the aforementioned connections. The MR estimates included in the meta-analysis did not show any heterogeneity according to the Cochran *Q* test, which assesses SNP estimates for heterogeneity. All Scatter plots and Leave-one-out plots were depicted in Supplementary material 2. This research excluded pleiotropy, as estimates exhibiting strong pleiotropy were eliminated. However, the meta-analysis for *Roseburia* and *Turicibacter* revealed significant heterogeneity. Figure 4 displays all combined estimates.

### 3.3 Reverse MR analysis

**Figure 5:**
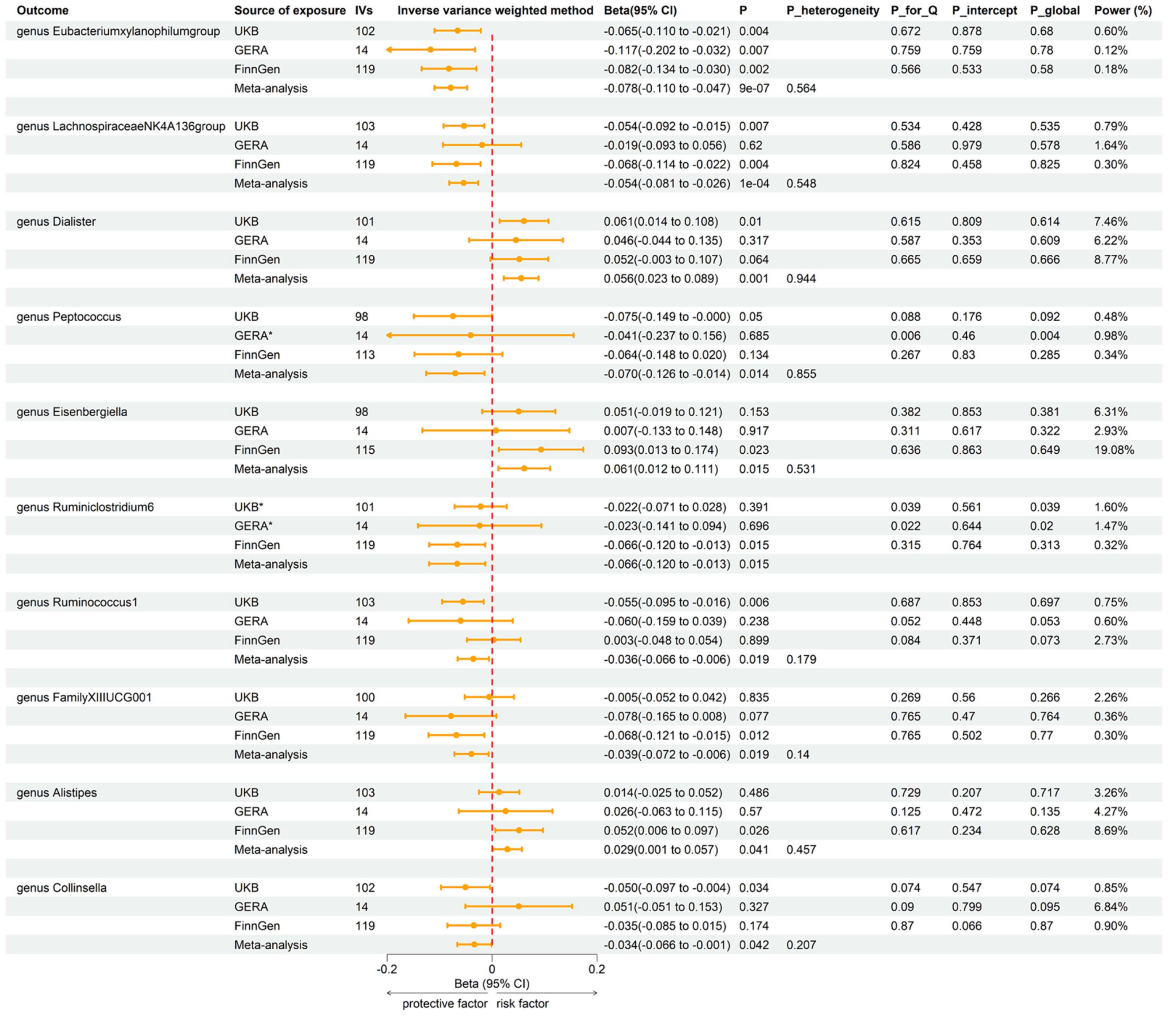
Forest plot of associations in reverse MR analysis. IVs, instrumental variables; CI, confident interval; *P*_heterogeneity, *p*-value of heterogeneity for meta-analysis; *P*_Q, *p*-value for Cochran *Q* test; *P*_intercept, *p*-value for MR-Egger intercept test; *P*_global, *p*-value for Global test; *, excluded from the meta-analysis due to SNPs less than 4 or significant pleiotropy.

**Figure 6:**
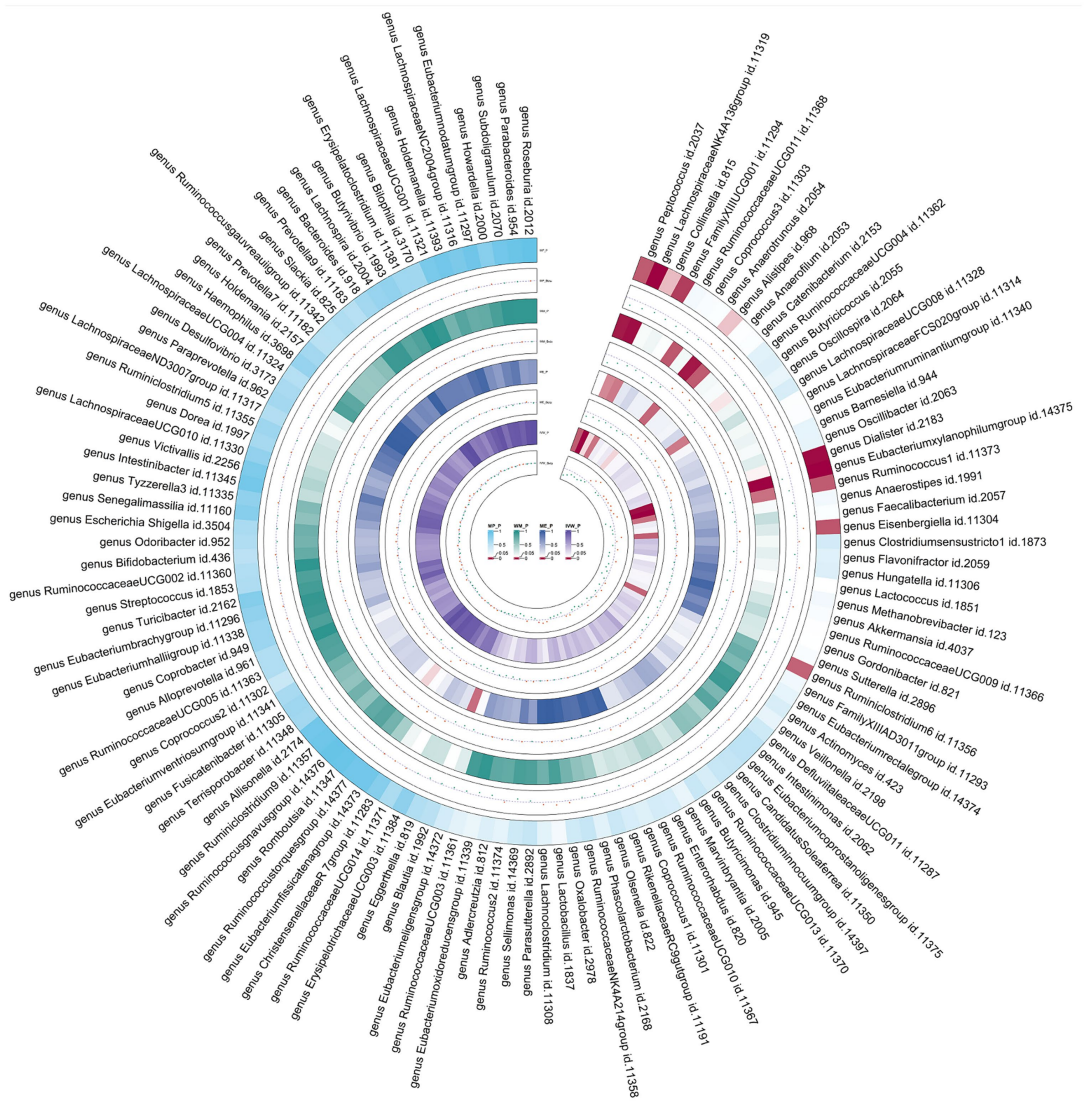
Circular heat map of meta-analysis of reverse MR analysis between gut microbiota and asthma. IVW, Inverse-Variance Weighted; ME, MR-Egger; WM, Weighted median; MP, MR-PRESSO. The color variations represented the size of the *p*-value. The scatter plots reflect Beta, with Beta > 0 labeled red and Beta < 0 labeled green.

Employing the same instrumental variables selection as for gut microbiota, 119 meta-analyses indicated significant associations between asthma and three bacterial genera, with suggestive associations for seven additional genera (Supplementary Table 4). The IVW method showed that genetically predicted asthma was associated with a significant decrease in the abundance of *Eubacteriumxylanophilumgroup* (Beta = −0.08, 95% CI −0.11 to −0.05; *p* = 9.25 × 10^−7^) and *LachnospiraceaeNK4A136group* (Beta = −0.05, 95% CI −0.08 to −0.03; *p* = 1.26 × 10^−4^), and an increase in the abundance of *Eisenbergiella* (Beta = 0.06, 95% CI 0.01–0.11; *p* = 0.015, Rg_*P* = 0.043). Suggestive negative associations were found with *Ruminococcus1*, *Collinsella*, *FamilyXIIIUCG001*, *Ruminiclostridium6*, and *Peptococcus*, and suggestive positive associations with *Dialister* and *Alistipes* (Figure 5). These findings were consistent across all sensitivity analyses, with no detected heterogeneity or pleiotropy. No heterogeneity was detected in the aggregated MR estimates within the meta-analysis using the Cochran *Q* test. The majority of the findings showed either no heterogeneity or only a slight amount. Figure 6 displays the collective estimates. Additionally, the bidirectional MR analysis did not find any indication of a two-way causal relationship between gut microbiota and asthma.

## 4 Discussion

Our comprehensive meta-analysis combining LDSC and MR presents novel insights into the genetic associations between specific gut microbiota and asthma. The findings from this study underscore a significant genetic correlation between certain gut microbiota genera, such as *RuminococcaceaeUCG004* and *Subdoligranulum*, and asthma, suggesting a potential role of these bacteria in the pathophysiology of asthma. Additionally, our analysis provides evidence for a causal relationship between several gut microbiota genera and asthma, highlighting the potential of gut microbiota as both a marker and a modulator of asthma risk.

In line with our analyses, research has shown that gut microbiota, including butyrate-producing bacteria like *Butyricicoccus*, *Butyrivibrio*, *RuminococcaceaeUCG004*, and *Subdoligranulum* play a crucial role in maintaining gut barrier integrity and modulating immune responses. For instance, butyrate, an SCFA, has anti-inflammatory properties and supports the development of regulatory T cells, which are essential for maintaining immune tolerance. Disruptions in the balance of butyrate-producing bacteria have been associated with various inflammatory conditions, including inflammatory bowel diseases and possibly asthma (Smith et al., 2013; Louis et al., 2014; Depner et al., 2020; Wan et al., 2023).

While SCFAs produced by certain gut bacteria are known for their anti-inflammatory effects, the relationship between gut microbiota and asthma is likely influenced by a multitude of factors beyond SCFAs alone (Di Cicco et al., 2018; Wang et al., 2018). Some bacteria, such as *Roseburia*, are SCFA producers yet are associated with an increased risk of asthma, indicating that other pathways may also be involved (Rutting et al., 2019; Singh et al., 2023). In addition to SCFAs, these bacteria may influence asthma through alternative pathways, such as modulation of the host’s immune system, interaction with other microbial metabolites, or through complex host-microbiota interactions influenced by genetic and environmental factors (Rutting et al., 2019; Lee-Sarwar et al., 2020; Singh et al., 2023). Future studies focusing on these complex interactions are warranted to better understand the multifaceted role of gut microbiota in asthma.

Bile acids, traditionally known for their role in digestion and absorption of fats, have emerged as significant immune modulators. They can influence the immune system through various receptors, including the farnesoid X receptor and the G protein-coupled bile acid receptor, which are expressed on immune cells. Activation of these receptors by bile acids can lead to anti-inflammatory effects and modulation of metabolic pathways. Turicibacter’s ability to modify bile acid profiles may impact the activation of these receptors, potentially leading to an environment less conducive to the development of allergic diseases like asthma. In addition, alterations in lipid metabolism, influenced by gut microbiota, including *Turicibacter*, can affect the body’s inflammatory status (Sayin et al., 2013). Lipids serve as energy sources, structural components of cell membranes, and signaling molecules. Dysregulation of lipid metabolism can lead to altered production of pro-inflammatory or anti-inflammatory lipid mediators, such as eicosanoids, which are derived from fatty acids and have been implicated in asthma pathogenesis. By modulating host lipid profiles, *Turicibacter* could influence the production of these mediators, potentially reducing inflammatory responses associated with asthma (Norris and Dennis, 2012).

In the Multiethnic Cohort-Adiposity Phenotype Study, *Coprococcus2* was indirectly linked to chronic low-grade systemic inflammation caused by diet and ectopic fat (Lozano et al., 2022). There is increasing evidence that asthma is associated with persistent low-grade inflammation, indicated by higher levels of inflammatory markers such as IL-4, IL-5, and IL-13 (Lambrecht and Hammad, 2015). This study is the first to report a connection between asthma and *Coprococcus2*. This finding could provide new insights into how dietary modifications that reduce *Coprococcus2* abundance might improve asthma outcomes.

Gut bacteria produce a diverse array of bioactive factors and metabolites, which can influence host physiology and disease risk through multiple pathways. These include not only SCFAs but also other bioactive compounds such as bile acids, tryptophan metabolites, and peptidoglycans. These compounds can modulate immune development and function, potentially influencing the risk of asthma. For example, some bacterial species produce metabolites that enhance the maturation of regulatory T cells, which are crucial for maintaining immune tolerance, while others produce factors that exacerbate inflammatory responses (Smith et al., 2013; Louis et al., 2014). Within the same genus, different species or subspecies of gut bacteria may have varying impacts on asthma risk, depending on the specific bioactive compounds they produce. For instance, the genus Bacteroides includes species that produce anti-inflammatory SCFAs, while others may produce metabolites that promote pro-inflammatory pathways (Depner et al., 2020). These differences highlight the importance of considering specific bacterial species or subspecies when evaluating the role of gut microbiota in asthma. Moreover, variations in the production of bioactive metabolites within a species could lead to different effects on the host’s immune system and asthma risk (Gu et al., 2022). The interaction between gut microbiota-derived bioactive factors and host pathways is likely multifactorial. SCFAs, for instance, promote the differentiation of regulatory T cells and modulate inflammatory responses (Smith et al., 2013). However, other bacterial metabolites, such as tryptophan metabolites, can influence immune responses by interacting with the aryl hydrocarbon receptor, while bacterial peptidoglycans and lipopolysaccharides engage toll-like receptors on host immune cells, activating signaling pathways that may affect asthma risk (Louis et al., 2014). These interactions suggest that the effects of gut bacteria on asthma are mediated through complex, indirect pathways involving multiple steps of modulation (Barcik et al., 2020; Song et al., 2024).

The gastrointestinal tract and respiratory tract (including oral and nasopharyngeal cavity), although separate organs, are part of a shared mucosal immune system termed the GLA. Airway colonization with pathogenic bacteria in early life is associated with an increased risk of respiratory allergic conditions (Bisgaard et al., 2023). The oral and nasopharyngeal microbiomes are often dominated by bacteria such as *Streptococcus*, *Neisseria*, *Prevotella*, *Rothia*, and *Haemophilus*. These microbiomes are crucial in the upper airways and can impact the immune responses in both the upper and lower respiratory tracts. For instance, the presence of pathogenic bacteria in these regions, especially during early life, has been associated with an increased risk of developing asthma. Pathogens like *Streptococcus pneumoniae* and *Haemophilus influenzae* in the nasopharynx have been linked to severe wheezing episodes in children and are predictors of asthma (Pérez-Losada et al., 2023). The respiratory microbiome extends from the nasopharynx into the lower respiratory tract and lungs. In asthmatic individuals, this microbiome often shows increased bacterial burden and reduced diversity, with a dominance of potentially pathogenic bacteria like *Haemophilus*, *Moraxella*, and *Neisseria*. These changes are associated with airway inflammation and exacerbations, particularly in patients with neutrophilic asthma, which is often more severe and less responsive to standard treatments like corticosteroids (Campbell et al., 2023). The gut microbiome plays a critical role in shaping the immune system, including the immune responses in the lungs, through what is known as the GLA. Dysbiosis in the gut microbiome, characterized by reduced diversity and an imbalance of beneficial bacteria like *Akkermansia* and *Faecalibacterium*, has been linked to increased susceptibility to asthma. SCFAs produced by gut bacteria, such as butyrate, have anti-inflammatory properties that can protect against asthma by enhancing the integrity of the gut barrier and modulating immune responses (Song et al., 2024). Rather than being direct causal agents of asthma, gut bacteria could also act as modulators or exacerbators of the immune responses initiated by the oral, nasopharyngeal, or respiratory microbiomes. For instance, an unhealthy gut microbiome could lead to a weakened immune response or promote systemic inflammation, which could exacerbate the inflammatory responses triggered by pathogens in the respiratory tract. Conversely, a healthy gut microbiome producing sufficient SCFAs might suppress these adverse responses, thereby reducing the severity or frequency of asthma exacerbations.

Although our study described a gut bacterial profile causally linked with the risk of asthma, some of the highlighted genera have also been linked with other systemic and respiratory illnesses. For example, *RuminococcaceaeUCG004* and *Subdoligranulum* were found to have a negative genetic correlation with asthma, suggesting a protective role, but they are also involved in gut health and have been linked to other conditions such as inflammatory bowel diseases (Gu et al., 2022). *Coprococcus2* have been implicated in conditions such as systemic inflammation and metabolic disorders (Lozano et al., 2022). Although direct evidence is lacking regarding the impact of the flora we identified on human respiratory diseases, it is reasonable to infer that their effects are not confined to asthma but extend to the entire system.

While our study primarily identified associations and potential causal links between specific gut bacteria and asthma, the interpretation of these changes should consider the context of disease progression. Changes in bacterial levels observed in patients with a prolonged history of asthma may represent an ongoing effort by the microbiome to counteract chronic inflammation or other pathological processes. This perspective highlights the importance of longitudinal studies that track microbiome changes over time in relation to disease progression and treatment responses. Future research should aim to distinguish between microbial shifts that are detrimental versus those that are protective or adaptive in chronic conditions like asthma.

Our research offers several advantages. First, the primary benefit is the MR design, which minimizes reverse causality and confounding factors (Burgess and Thompson, 2015). Furthermore, we mitigated the potential influence of population structure bias by primarily studying individuals of European descent. However, it is important to note that this approach may limit the applicability of our results to other ethnic groups. We used meta-analysis to bolster the robustness of our results, reducing any fluctuations that might arise from relying on a single database. Additionally, we enhanced the reliability of our findings by excluding MR estimates influenced by substantial pleiotropy from our meta-analysis. Finally, we used the LDSC correlation *p*-values method to lower the rate of false negatives and implemented Bonferroni’s correction to minimize the rate of false positives associated with multiple analyses.

It is important to recognize the limitations of our research to properly evaluate its findings. First, the GWAS data on gut microbiota were collected from a heterogeneous group of 18,340 individuals from various ethnicities. However, the GWAS summary findings for asthma were derived solely from individuals of European ancestry. This disparity might limit the applicability of our findings to other ethnic and demographic cohorts. While nearly 80% of the data on gut microbiota comes from people of European descent, additional research involving a more diverse spectrum of populations is needed to validate our results and ensure their broader relevance. Second, although MR methods help reduce confounding and reverse causation, potential biases still exist. For instance, population stratification can introduce bias if there are systematic differences in allele frequencies between subpopulations. We mitigated this by focusing on individuals of European ancestry, but residual confounding may still influence our results. Additionally, pleiotropy, where genetic variants influence multiple traits, could bias the causal estimates. We used MR-Egger and MR-PRESSO to detect and adjust for pleiotropy, but these methods have limitations and may not entirely eliminate pleiotropic effects. Third, the power estimates for our overall MR analyses are relatively low. This low power could potentially lead to false-negative results where true associations may not be detected. The complexity of the genetic architecture underlying gut microbiota and asthma interactions may require even larger sample sizes or more refined genetic instruments to adequately capture the causal relationships. Fourth, our ability to investigate potential disparities across different demographics was limited by the lack of detailed data, which prevented stratified analysis by age and gender. Finally, although our rigorous study design helped identify some causal relationships, much remains to be learned about the pathophysiology and complexity of the gut microbiota. This underscores the need for further research to clarify this field.

## 5 Conclusion

In conclusion, our study provides robust evidence of a causal relationship between specific gut microbiomes and asthma, laying a foundation for future research into gut microbiota-targeted therapies. These findings open new avenues for exploring the gut microbiota’s role in asthma and developing novel preventative and therapeutic strategies for managing this complex condition.

## Data Availability

The original contributions presented in the study are included in the article/Supplementary material, further inquiries can be directed to the corresponding author.
