# Supplementary material for: Unveiling genetic links between gut microbiota and asthma: a Mendelian randomization
Source: Front Microbiol. 2024 Sep 20;15:1448629. doi: 10.3389/fmicb.2024.1448629 (PMC11449699; doi:10.3389/fmicb.2024.1448629)
Supplement: Supplementary file 1 [file Data_Sheet_1.PDF]

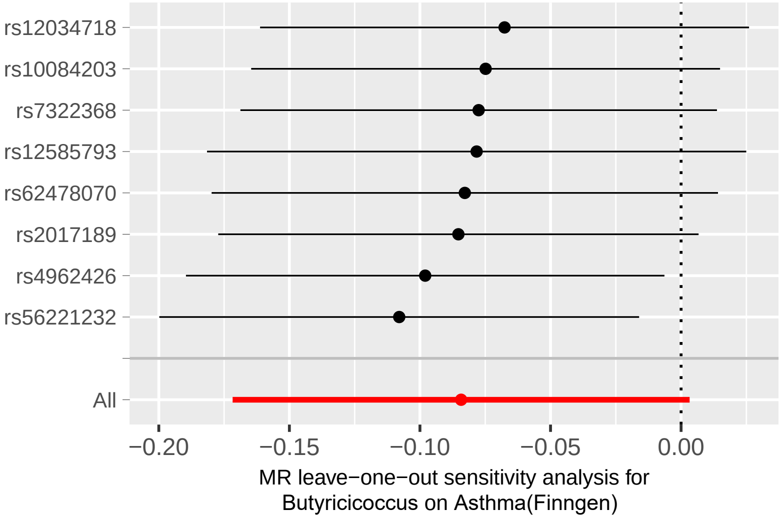
rs12034718
rs10084203
rs7322368
rs12585793
rs62478070
rs2017189
rs4962426
rs56221232
All
−0.20
−0.15
−0.10
−0.05
0.00
MR leave−one−out sensitivity analysis for
Butyricicoccus on Asthma(Finngen)

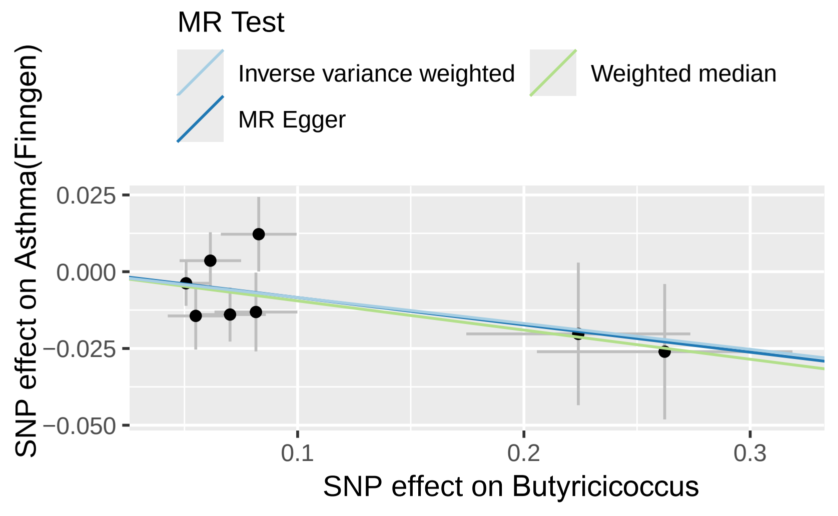
MR Test
Inverse variance weighted
Weighted median
MR Egger
SNP effect on Asthma(Finngen)
0.025
0.000
−0.025
−0.050
0.1
0.2
0.3
SNP effect on Butyricicoccus

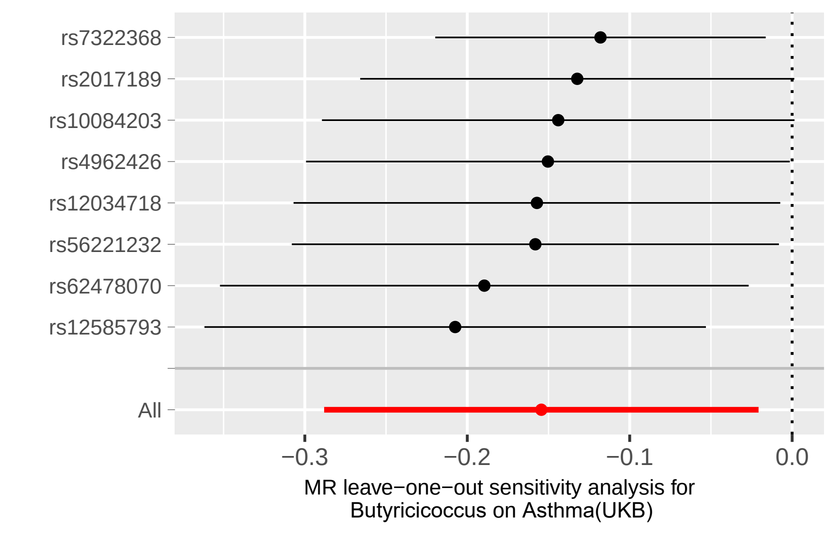
rs7322368
rs2017189
rs10084203
rs4962426
rs12034718
rs56221232
rs62478070
rs12585793
All
−0.3
−0.2
−0.1
0.0
MR leave−one−out sensitivity analysis for
Butyricicoccus on Asthma(UKB)

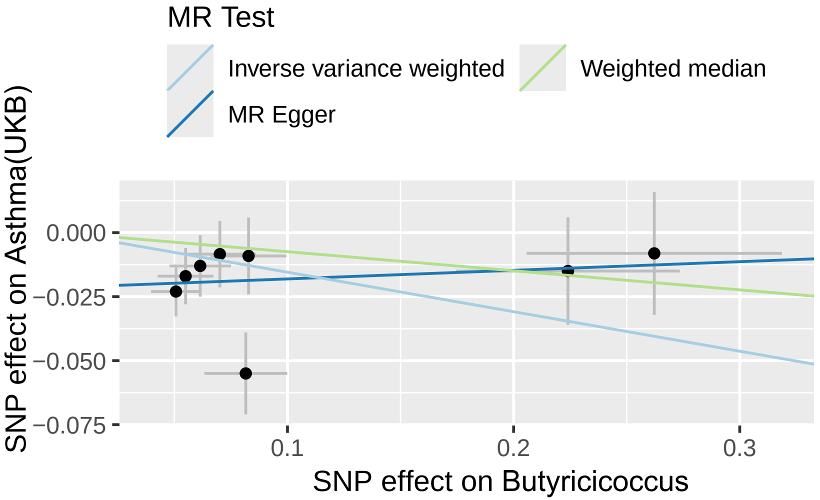

MR Test
Inverse variance weighted
Weighted median
MR Egger
SNP effect on Asthma(UKB)
0.000
−0.025
−0.050
−0.075
0.1
0.2
0.3
SNP effect on Butyricicoccus

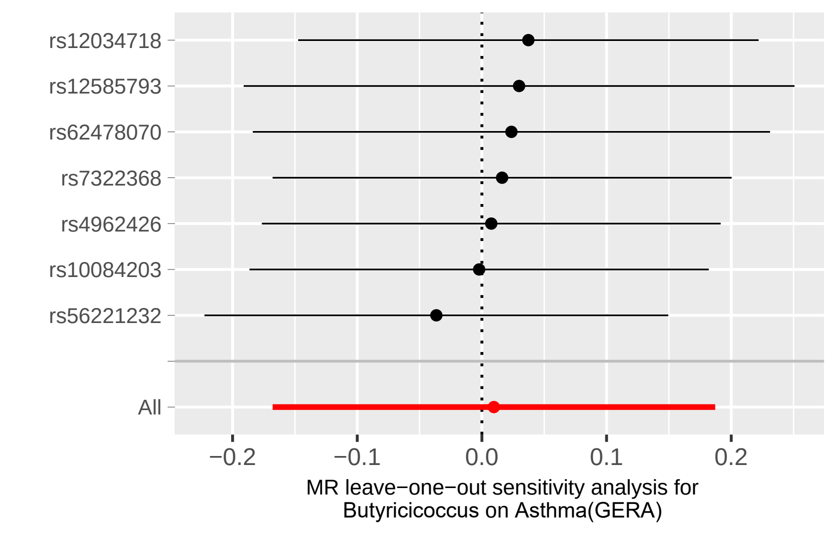
rs12034718
rs12585793
rs62478070
rs7322368
rs4962426
rs10084203
rs56221232
All
−0.2
−0.1
0.0
0.1
0.2
MR leave−one−out sensitivity analysis for
Butyricicoccus on Asthma(GERA)

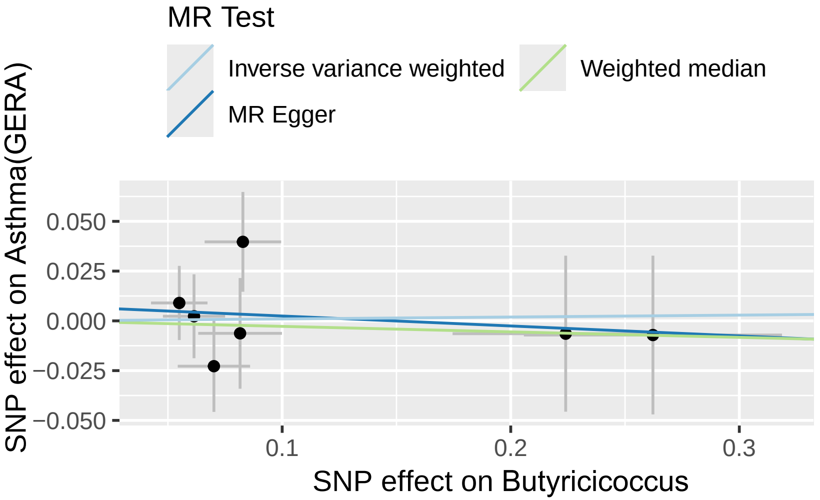

MR Test
Inverse variance weighted
Weighted median
MR Egger
SNP effect on Asthma(GERA)
0.050
0.025
0.000
−0.025
−0.050
0.1
0.2
0.3
SNP effect on Butyricicoccus

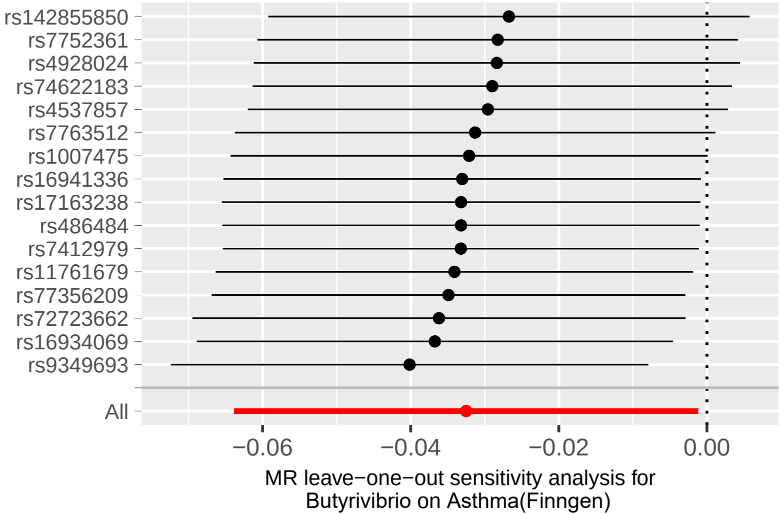
rs142855850
rs7752361
rs4928024
rs74622183
rs4537857
rs7763512
rs1007475
rs16941336
rs17163238
rs486484
rs7412979
rs11761679
rs77356209
rs72723662
rs16934069
rs9349693
All
−0.06
−0.04
−0.02
0.00
MR leave−one−out sensitivity analysis for
Butyrivibrio on Asthma(Finngen)

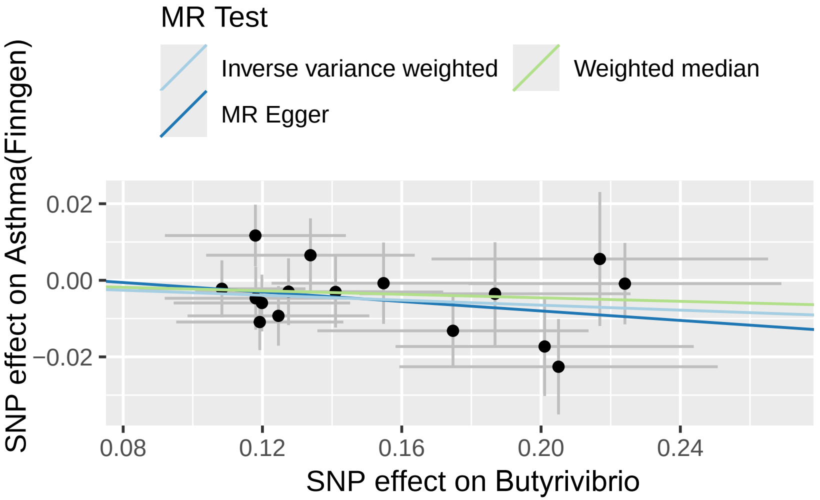

MR Test
Inverse variance weighted
Weighted median
MR Egger
SNP effect on Asthma(Finngen)
0.02
0.00
−0.02
0.08
0.12
0.16
0.20
0.24
SNP effect on Butyrivibrio

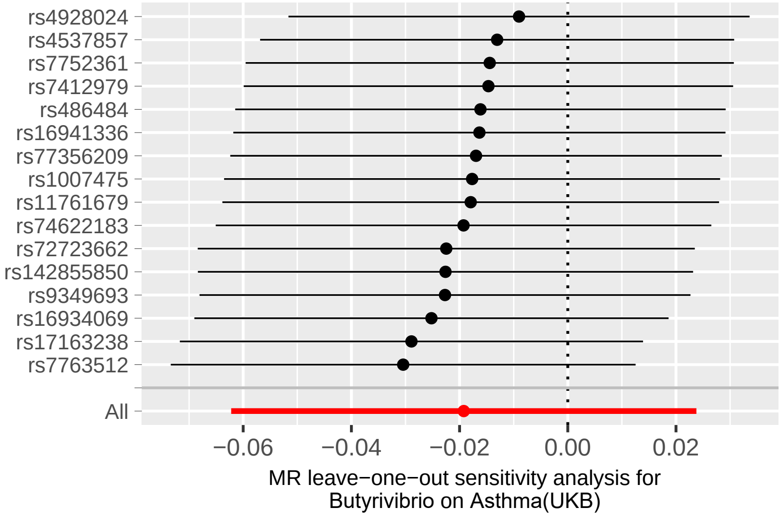
rs4928024
rs4537857
rs7752361
rs7412979
rs486484
rs16941336
rs77356209
rs1007475
rs11761679
rs74622183
rs72723662
rs142855850
rs9349693
rs16934069
rs17163238
rs7763512
All
−0.06
−0.04
−0.02
0.00
0.02
MR leave−one−out sensitivity analysis for
Butyrivibrio on Asthma(UKB)

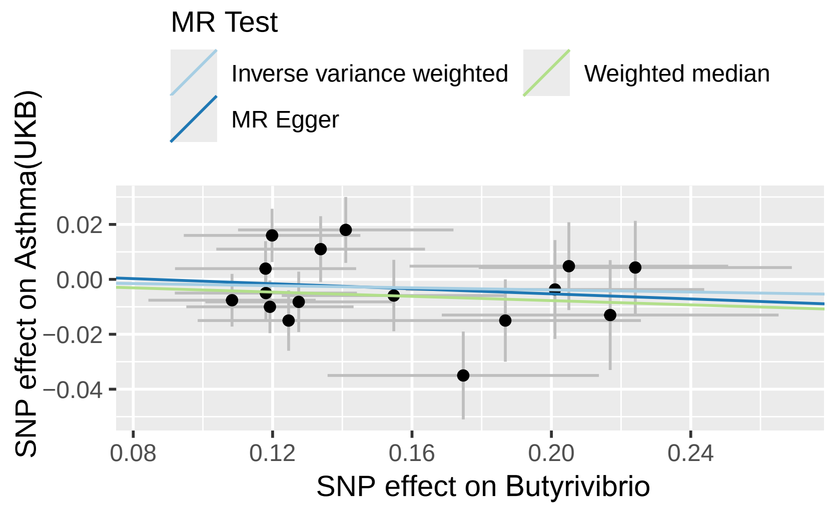
MR Test
Inverse variance weighted
MR Egger
Weighted median
SNP effect on Asthma(UKB)
0.02
0.00
−0.02
−0.04
0.08
0.12
0.16
0.20
0.24
SNP effect on Butyrivibrio

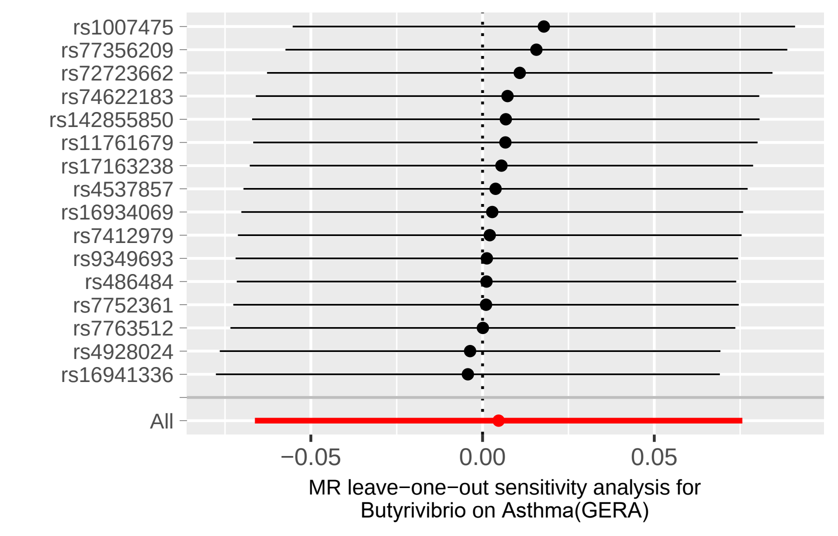
rs1007475
rs77356209
rs72723662
rs74622183
rs142855850
rs11761679
rs17163238
rs4537857
rs16934069
rs7412979
rs9349693
rs486484
rs7752361
rs7763512
rs4928024
rs16941336
All
−0.05
0.00
0.05
MR leave−one−out sensitivity analysis for
Butyrivibrio on Asthma(GERA)

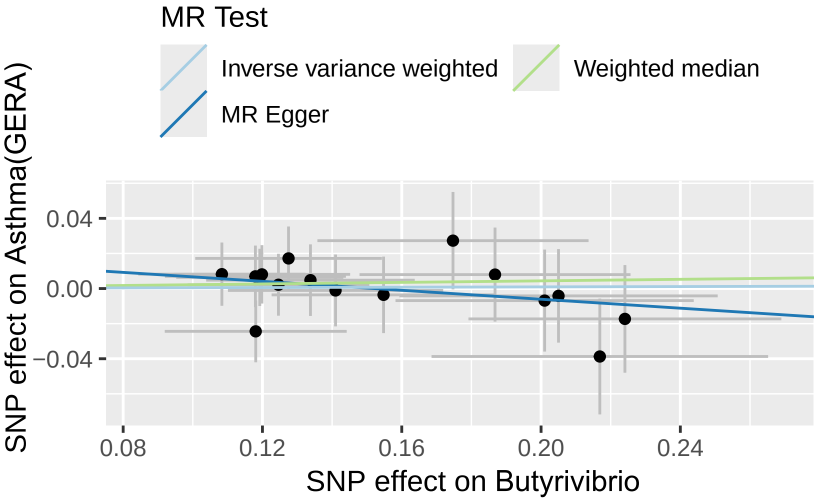
MR Test
Inverse variance weighted
MR Egger
Weighted median
SNP effect on Asthma(GERA)
0.04
0.00
−0.04
0.08
0.12
0.16
0.20
0.24
SNP effect on Butyrivibrio

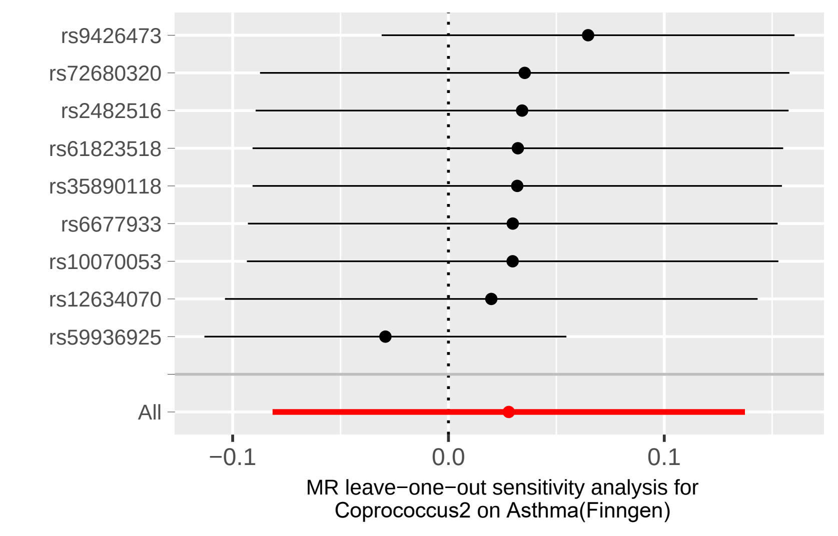
rs9426473
rs72680320
rs2482516
rs61823518
rs35890118
rs6677933
rs10070053
rs12634070
rs59936925
All
−0.1
0.0
0.1
MR leave−one−out sensitivity analysis for
Coprococcus2 on Asthma(Finngen)

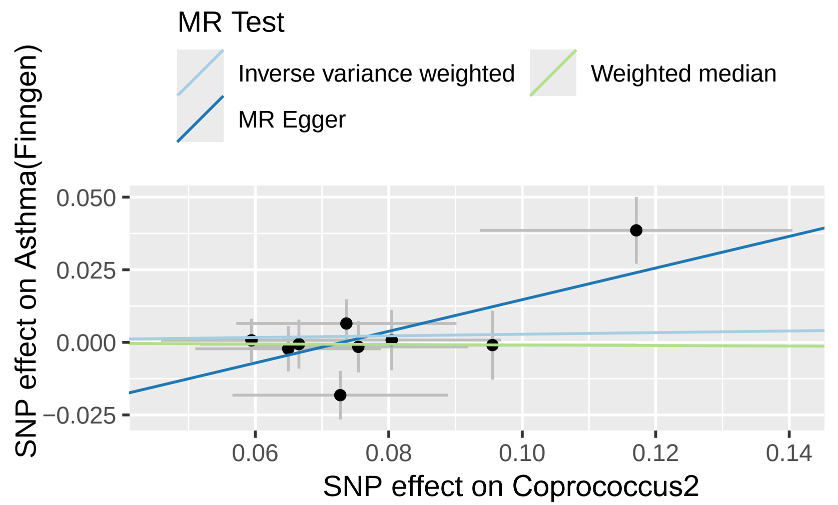
MR Test
Inverse variance weighted
Weighted median
MR Egger
SNP effect on Asthma(Finngen)
0.050
0.025
0.000
−0.025
0.06
0.08
0.10
0.12
0.14
SNP effect on Coprococcus2

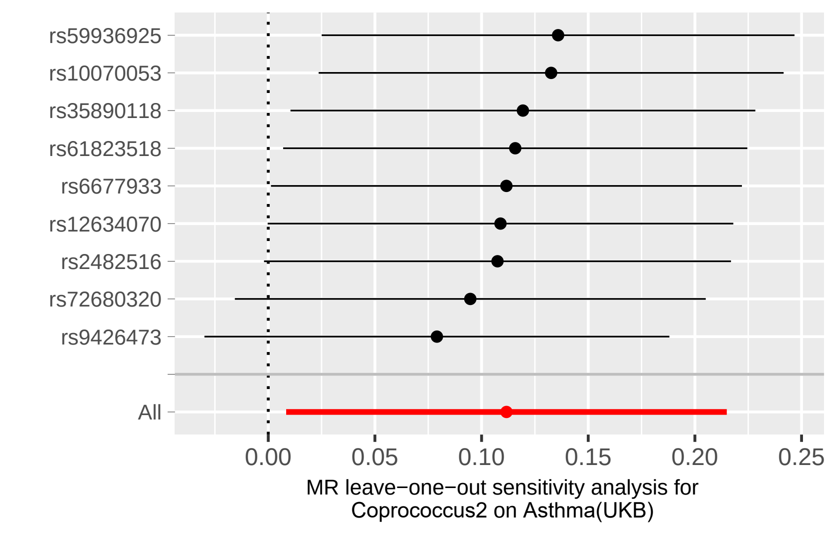

rs59936925
rs10070053
rs35890118
rs61823518
rs6677933
rs12634070
rs2482516
rs72680320
rs9426473
All
0.00
0.05
0.10
0.15
0.20
0.25
MR leave−one−out sensitivity analysis for
Coprococcus2 on Asthma(UKB)

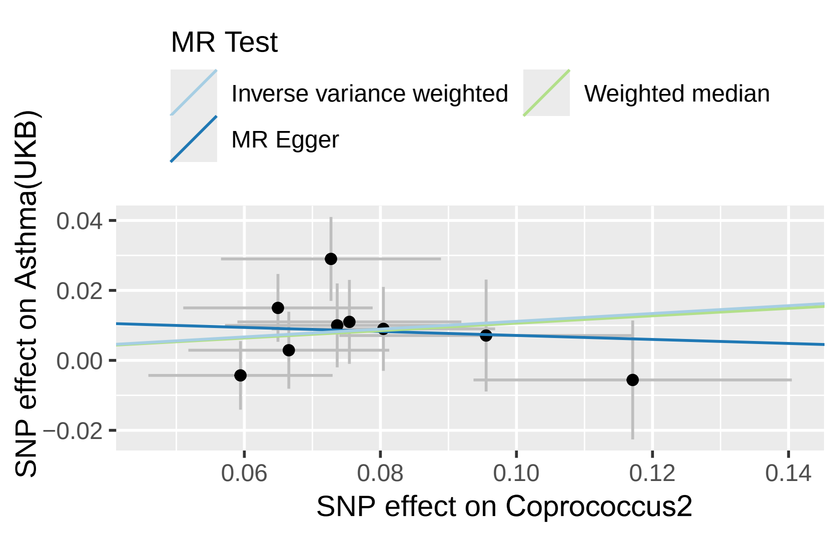

MR Test
Inverse variance weighted
Weighted median
MR Egger
SNP effect on Asthma(UKB)
0.04
0.02
0.00
−0.02
0.06
0.08
0.10
0.12
0.14
SNP effect on Coprococcus2

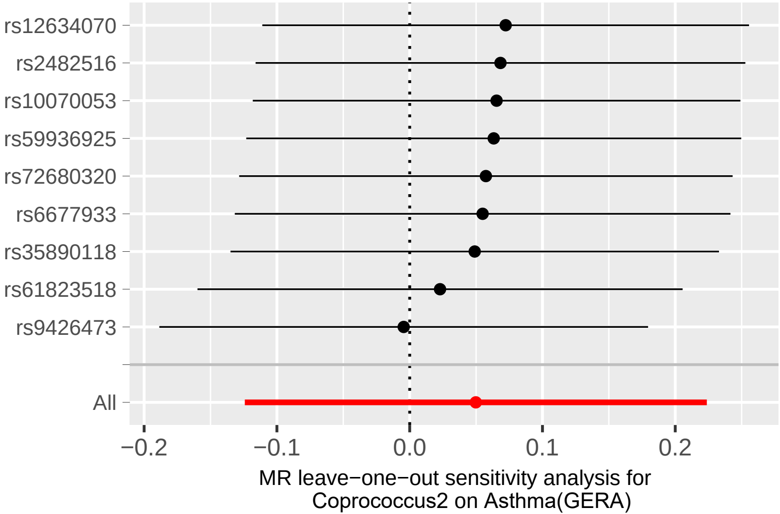
rs12634070
rs2482516
rs10070053
rs59936925
rs72680320
rs6677933
rs35890118
rs61823518
rs9426473
All
−0.2
−0.1
0.0
0.1
0.2
MR leave−one−out sensitivity analysis for
Coprococcus2 on Asthma(GERA)

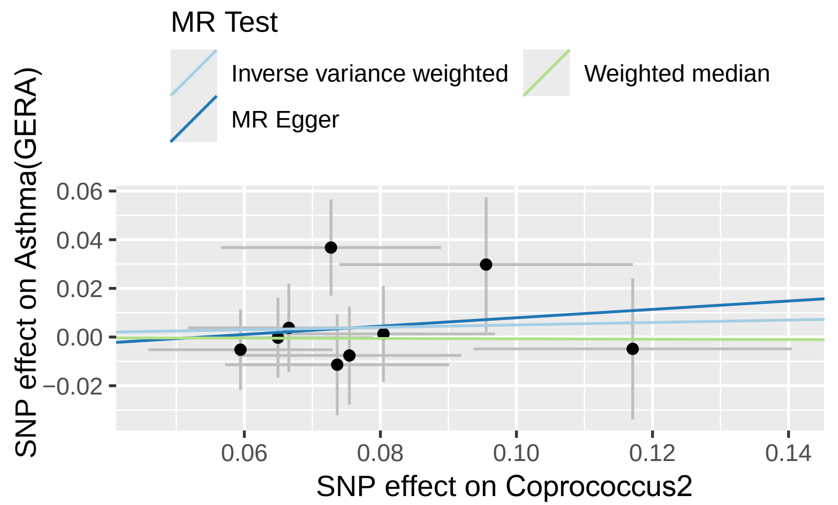

MR Test
Inverse variance weighted
MR Egger
Weighted median
0.06
0.04
0.02
0.00
−0.02
0.06
0.08
0.10
0.12
0.14
SNP effect on Asthma(GERA)
SNP effect on Coprococcus2

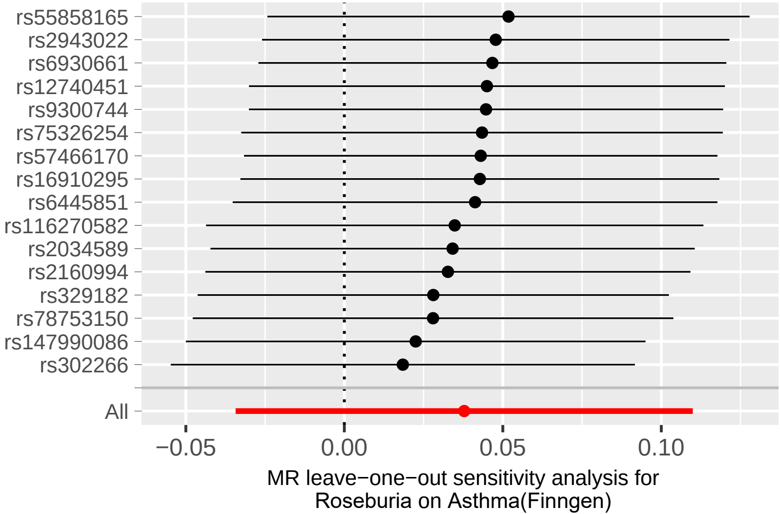

rs55858165
rs2943022
rs6930661
rs12740451
rs9300744
rs75326254
rs57466170
rs16910295
rs6445851
rs116270582
rs2034589
rs2160994
rs329182
rs78753150
rs147990086
rs302266
All
−0.05
0.00
0.05
0.10
MR leave−one−out sensitivity analysis for
Roseburia on Asthma(Finngen)

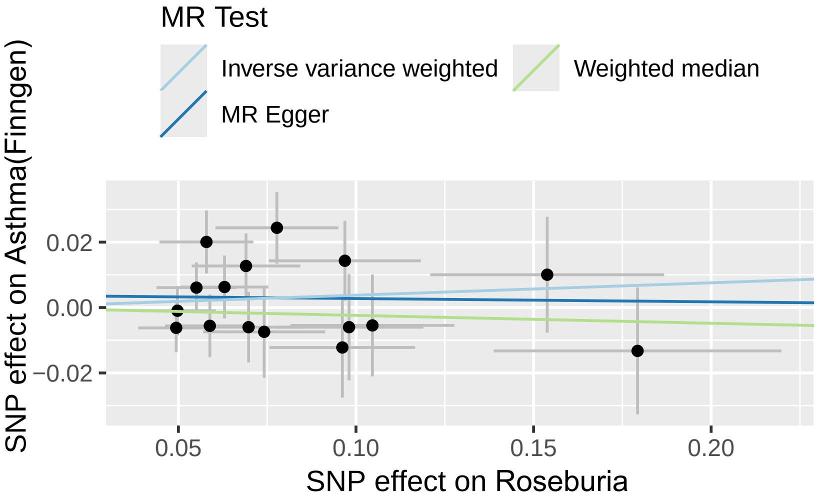
MR Test
Inverse variance weighted
MR Egger
Weighted median
SNP effect on Asthma(Finngen)
0.02
0.00
−0.02
0.05
0.10
0.15
0.20
SNP effect on Roseburia

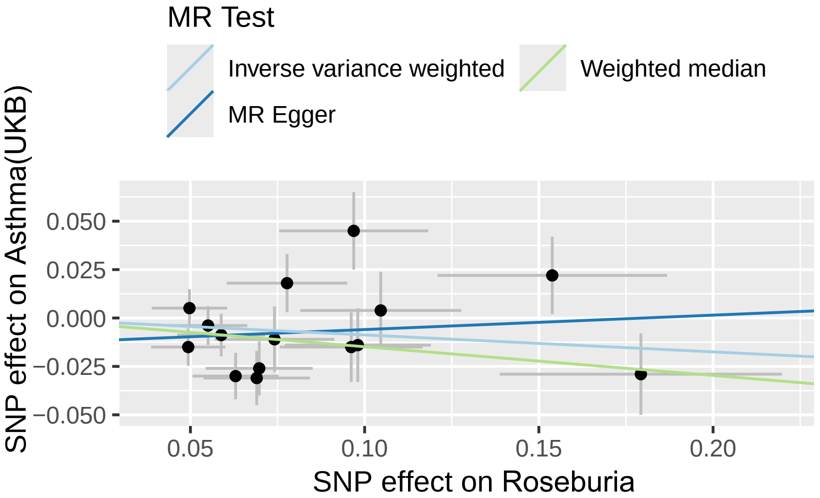
MR Test
Inverse variance weighted
MR Egger
Weighted median
SNP effect on Asthma(UKB)
0.050
0.025
0.000
−0.025
−0.050
0.05
0.10
0.15
0.20
SNP effect on Roseburia

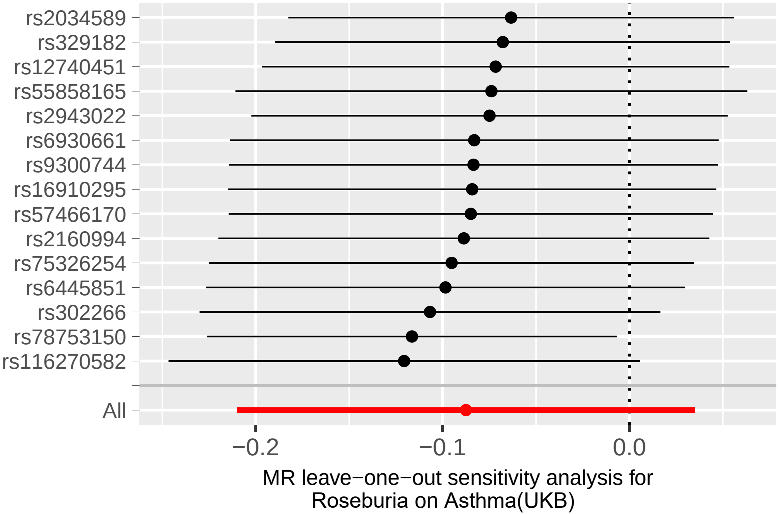
rs2034589
rs329182
rs12740451
rs55858165
rs2943022
rs6930661
rs9300744
rs16910295
rs57466170
rs2160994
rs75326254
rs6445851
rs302266
rs78753150
rs116270582
All
−0.2
−0.1
0.0
MR leave−one−out sensitivity analysis for
Roseburia on Asthma(UKB)

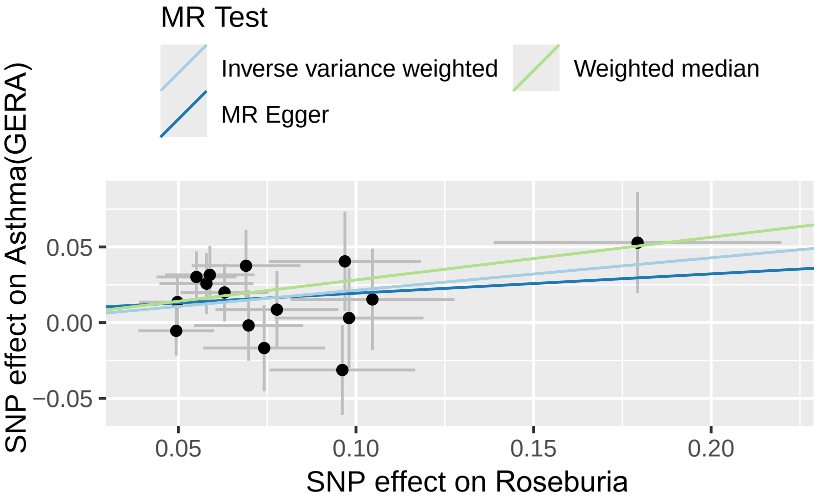
MR Test
Inverse variance weighted
Weighted median
MR Egger
SNP effect on Asthma(GERA)
0.05
0.00
−0.05
0.05
0.10
0.15
0.20
SNP effect on Roseburia

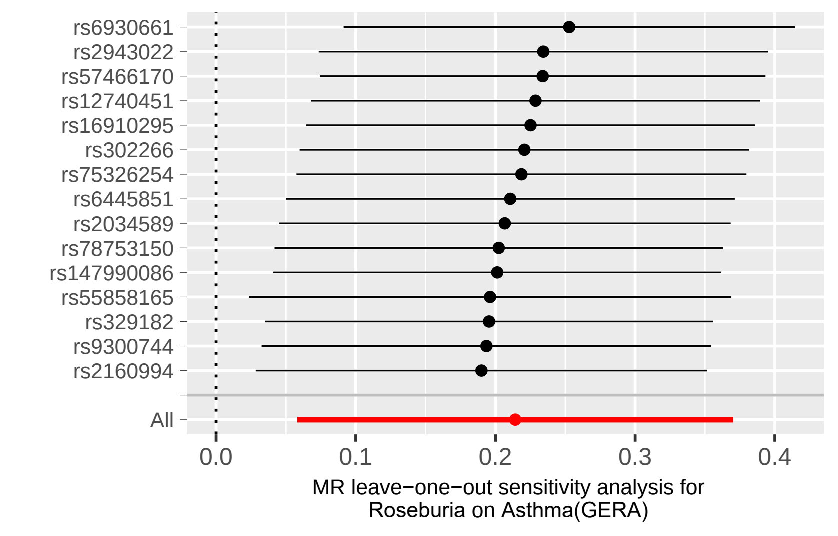
rs6930661
rs2943022
rs57466170
rs12740451
rs16910295
rs302266
rs75326254
rs6445851
rs2034589
rs78753150
rs147990086
rs55858165
rs329182
rs9300744
rs2160994
All
0.0
0.1
0.2
0.3
0.4
MR leave−one−out sensitivity analysis for
Roseburia on Asthma(GERA)

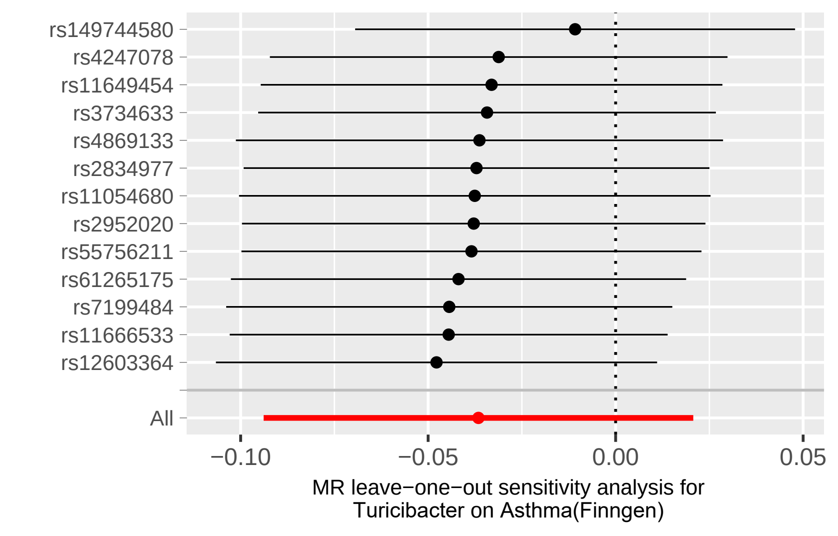
rs149744580
rs4247078
rs11649454
rs3734633
rs4869133
rs2834977
rs11054680
rs2952020
rs55756211
rs61265175
rs7199484
rs11666533
rs12603364
All
−0.10
−0.05
0.00
0.05
MR leave−one−out sensitivity analysis for
Turicibacter on Asthma(Finngen)

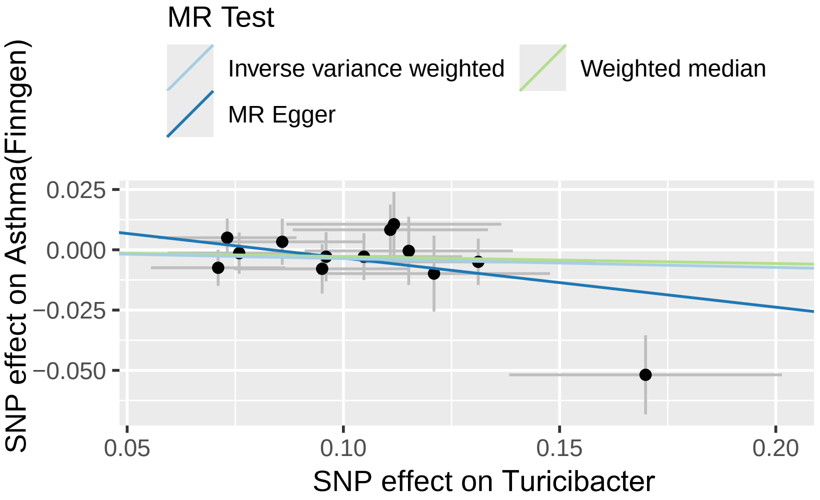
MR Test
Inverse variance weighted
Weighted median
MR Egger
SNP effect on Asthma(Finngen)
0.025
0.000
−0.025
−0.050
0.05
0.10
0.15
0.20
SNP effect on Turicibacter

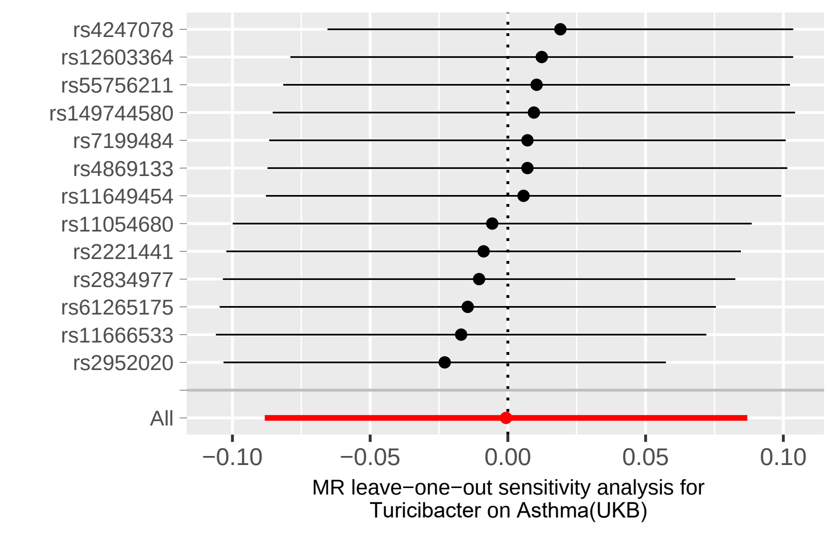
rs4247078
rs12603364
rs55756211
rs149744580
rs7199484
rs4869133
rs11649454
rs11054680
rs2221441
rs2834977
rs61265175
rs11666533
rs2952020
All
−0.10
−0.05
0.00
0.05
0.10
MR leave−one−out sensitivity analysis for
Turicibacter on Asthma(UKB)

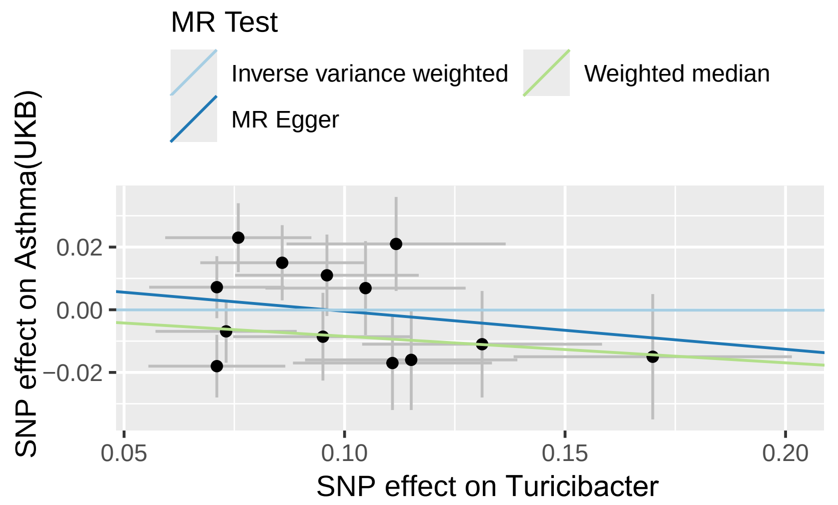
MR Test
Inverse variance weighted
MR Egger
Weighted median
SNP effect on Asthma(UKB)
0.02
0.00
−0.02
0.05
0.10
0.15
0.20
SNP effect on Turicibacter

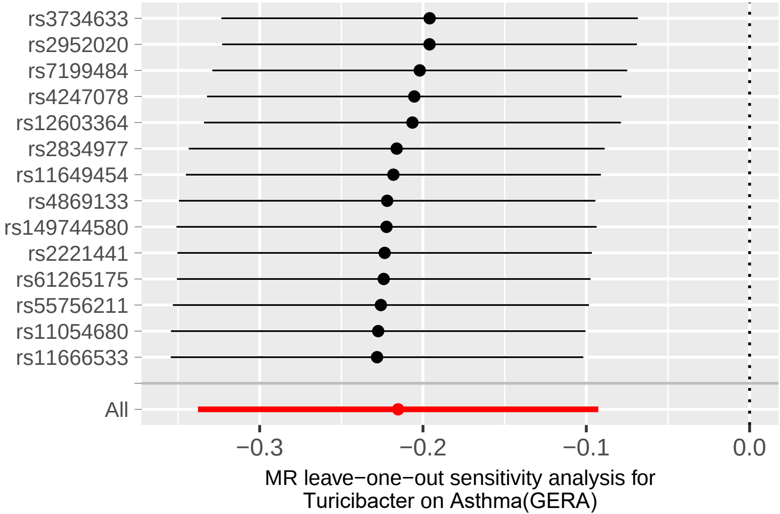
rs3734633
rs2952020
rs7199484
rs4247078
rs12603364
rs2834977
rs11649454
rs4869133
rs149744580
rs2221441
rs61265175
rs55756211
rs11054680
rs11666533
All
−0.3
−0.2
−0.1
0.0
MR leave−one−out sensitivity analysis for
Turicibacter on Asthma(GERA)

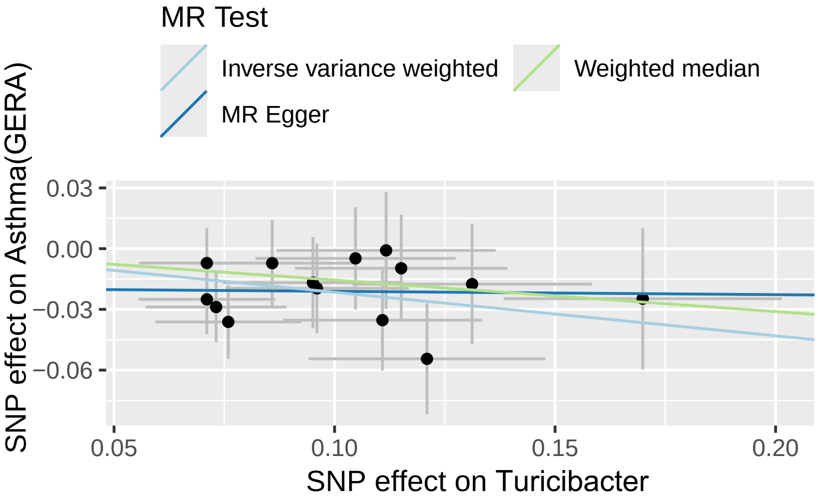
MR Test
Inverse variance weighted
Weighted median
MR Egger
SNP effect on Asthma(GERA)
0.03
0.00
−0.03
−0.06
0.05
0.10
0.15
0.20
SNP effect on Turicibacter
